# P02.161. A randomized, active-controlled trial of school-based mindfulness instruction for urban middle-school male youth

**DOI:** 10.1186/1472-6882-12-S1-P217

**Published:** 2012-06-12

**Authors:** E Sibinga, C Perry-Parrish, S Chung, S Johnson, M Smith, J Ellen

**Affiliations:** 1 Johns Hopkins School of Medicine, Baltimore, USA

## Purpose

Our research of MBSR for mostly female urban youth showed reductions in conflict, anxiety, and stress, as well as increased self-regulation. Reviews of mindfulness instruction suggest benefit but call for increased methodological rigor, particularly active control conditions. Here, we explore the specific effects of MBSR compared with an active control on stress and coping among young urban males.

## Methods

Participants were 7th and 8th grade boys in a small urban middle school for boys. They were randomly assigned to MBSR or an active control (Healthy Topics—HT), an age-appropriate health education program, designed to control for positive adult instructor, learning new information, and class time. Data were collected at baseline, post-program, and three-month follow-up on psychological symptoms, stress, mindfulness, coping; sleep; teacher-rated behavior; and salivary cortisol, a physiologic measure of stress.

## Results

Forty-one (22 MBSR and 19 HT) boys participated. Ninety-five percent were African American, with a mean age of 12.5 years. There were no significant differences at baseline between groups. Following the programs, MBSR boys had significantly less anxiety (p=0.01), less rumination (p=0.02), and less negative coping (p=0.06) than HT boys. From pre- to post-program, daily cumulative cortisol levels increased during the academic terms for HT participants at a trend level (p=0.07) but remained constant for MBSR participants (p=0.33). Otherwise, we did not detect differences in outcomes.

## Conclusion

This study of MBSR compared with an active control for urban male youth shows less rumination, anxiety, and an attenuation of cortisol increase among MBSR participants. These results suggest that MBSR specifically enhances self-regulatory processes for urban male youth, including improved coping and emotion regulation. Additional research is needed to explore the impact of mindfulness instruction for urban male youth on self-regulation, the duration of its effect, and related social, psychological, and behavioral outcomes.

